# New Appliances and Things Medical

**Published:** 1894-02-24

**Authors:** 


### NEW APPLIANCES AND THINGS MEDICAL.

[All preparations, appliances, novelties, &c., of which a notice is desired, should be sent for the Editor, to care of The Manager, 428, Strand, London, W.C.]

### CATALOGUE OF SURGICAL INSTRUMENTS AND DRUGGISTS' SUNDRIES.

Lynch and Co., Aldersgate Street, London, E.C.

Messrs. Lynch have forwarded us a copy of their catalogue of surgical instruments and druggists' appliances. It is not often that we have to record anything new in the arrangement or compilation of manufacturers' catalogues. In this instance, however, the above firm have introduced a new method of indexing their goods. The letterpress is quite distinct from the illustration but the two are bound up together and numbered to correspond, so that reference to both and a comparison between the two is rendered as easy as possible. This firm have a large supply of Lewis's metallic splints, for the general practitioner, perhaps, the most useful kind in the market, since though they are sufficiently malleable to allow manipulation into any shape, they are quite strong enough to prevent displacement in the fracture and of bones. In ordering splints and other surgical appliances for deformities, &c., the directions and diagrams given in the catalogue render mistakes highly improbable. Messrs. Lynch pay special attention to indiarubber goods. All their patterns of enemas are excellent, Lynch's "patent safety enema," for preventing the injection of air, is highly to be recommended, both for the administration of nutrient enemas and for general purposes.

